# Pagetoid reticulosis (Woringer–Kolopp disease) mimicking eczema: a case report with immunophenotypic analysis and literature review

**DOI:** 10.3389/fimmu.2026.1821015

**Published:** 2026-06-30

**Authors:** Yansi Lyu, Zonghua Wen, Jiefeng Jiang, Lyuxin Guan, Ziqin Gan, Maomao An, Suchun Hou

**Affiliations:** 1 Department of Dermatology, Shenzhen University General Hospital, Shenzhen, Guangdong, China; 2 Department of Pathology, Shenzhen University General Hospital, Shenzhen, Guangdong, China; 3 The First School of Clinical Medicine, Southern Medical University, Guangzhou, China; 4 Department of Burn and Plastic Surgery, The First Affiliated Hospital of Shenzhen University, Shenzhen, China; 5 Department of Dermatology, The Fifth Affiliated Hospital of Sun Yat-sen University, Zhuhai, China; 6 Department of Pharmacology, Shanghai Tenth People’s Hospital, Tongji University School of Medicine, Shanghai, China

**Keywords:** eczema, epidermotropism, mycosis fungoides, pagetoid reticulosis, Woringer–Kolopp disease

## Abstract

Pagetoid reticulosis (PR), also known as Woringer–Kolopp disease (WKD), is a rare subtype of cutaneous T-cell lymphoma. Due to its low incidence and non-specific clinical presentation, it is frequently prone to misdiagnosis. We report the case of a 53-year-old female with a 5-year history of a recalcitrant, pruritic erythematous plaque on the right ankle, initially mismanaged as chronic eczema. Clinical examination revealed a solitary, well-demarcated, infiltrated scaly plaque at an acral site. Histopathological analysis demonstrated striking epidermotropism of medium-sized atypical lymphocytes with a pagetoid distribution and Pautrier-like microabscesses. Immunohistochemistry (IHC) revealed a T-cell lineage (CD3+/CD5+) with significant loss of CD4, CD7, and predominantly absent CD2/CD8 expression. A Ki-67 proliferation index of approximately 40% and focal CD30 positivity were observed, while EBER *in situ* hybridization was negative. Following a definitive diagnosis of localized PR, the patient was treated with topical clobetasol propionate twice daily, resulting in substantial clinical resolution. Literature review highlights the marked immunophenotypic heterogeneity of WKD and its favorable prognosis in localized forms. For treatment-refractory acral plaques, a high index of clinical suspicion is paramount. Early biopsy with appropriate immunophenotypic evaluation is essential to avoid diagnostic delay and guide effective local treatment, including radiotherapy or surgical excision.

## Introduction

Pagetoid reticulosis (PR), alternatively designated as Woringer–Kolopp disease (WKD), represents a rare, localized clinicopathologic variant of mycosis fungoides (MF) (1). It typically manifests as a solitary, well-demarcated, hyperkeratotic plaque predominantly affecting acral sites in both pediatric and adult populations, characterized by a characteristically indolent and protracted clinical course (2). The condition exhibits a slight male predominance, with a male-to-female ratio of approximately 2:1 (3). Clinically, PR is stratified into localized and disseminated forms: the former presents as solitary erythema or plaques with a favorable prognosis, whereas the latter is exceedingly rare, presenting with multifocal lesions and carrying a comparatively less optimistic prognosis (4).

Due to its insidious progression and non-specific clinical morphology, PR frequently eludes definitive diagnosis for years (5). Its clinical phenotype closely mimics several inflammatory dermatoses, rendering it particularly prone to being mismanaged as chronic eczema or psoriasis, which consequently leads to recurrent symptoms and an unnecessary therapeutic burden (2, 6). However, upon timely diagnostic confirmation and appropriate intervention, the prognosis is generally excellent, with most patients achieving complete clinical remission (6).

The initial differential diagnosis of WKD encompasses localized psoriasis, contact dermatitis, eczematous dermatitis, and fungal or bacterial infections (1). Histopathologically, the disease is defined by epidermal hyperplasia (hyperkeratosis and acanthosis) accompanied by striking epidermotropic infiltration of atypical lymphocytes, typically arranged in a scattered or clustered “pagetoid” pattern (7). The superficial dermis commonly harbors a mild-to-moderate mixed inflammatory infiltrate, while neoplastic T-cell involvement within the dermis remains minimal (8). Given the rarity of this entity, current evidence is principally derived from case reports, small series, and retrospective analyses.

Localized Woringer–Kolopp disease remains an uncommon clinicopathologic entity with variable clinical and immunophenotypic features. Here, we report a case of localized WKD presenting as a solitary, well-demarcated, treatment-refractory acral plaque with an atypical immunophenotypic profile. Through a review of the relevant literature, we summarize the clinicopathological features, immunophenotypic variability, differential diagnosis, and treatment options of localized WKD.

## Clinical case

A 53-year-old woman presented to our department on August 21, 2025, for evaluation of a 5-year history of persistent pruritic erythema on the right ankle. The disease onset was insidious, characterized by a waxing and waning course accompanied by mild pruritus. Prior to her visit, multiple differential diagnoses, including eczema, erythema elevatum diutinum, and granuloma annulare, had been entertained at external institutions. Despite the topical application of 0.1% tacrolimus ointment, the therapeutic response remained suboptimal. The patient reported no arthralgia, alopecia, or other systemic manifestations. Her medical history was otherwise unremarkable, with no known drug or food allergies. In light of the refractory nature of the presumed “eczema,” a cutaneous neoplasm was suspected, prompting a diagnostic biopsy with ancillary studies.

**Figure 1:**
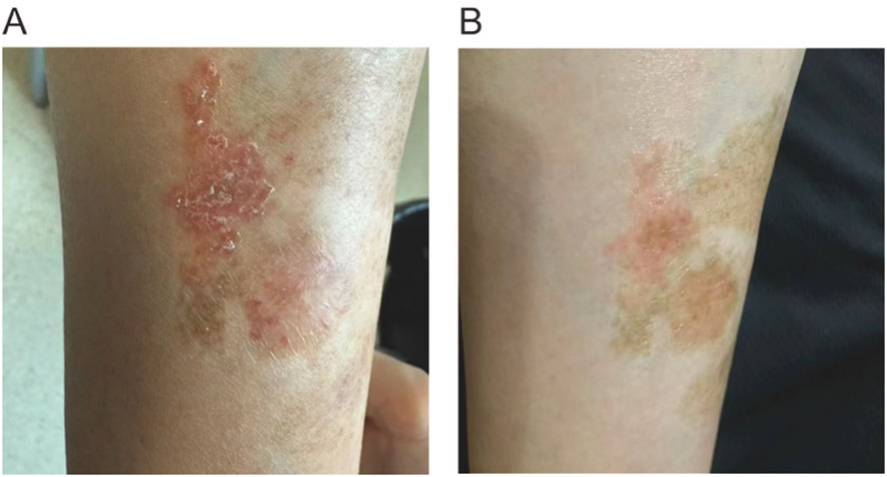
Clinical presentation of the right ankle lesion before and after treatment. **(A)** A solitary, well-demarcated, infiltrated erythematous plaque with overlying scale on the right ankle at initial presentation. **(B)** Substantial regression of the lesion after treatment with topical clobetasol propionate.

**Figure 2:**
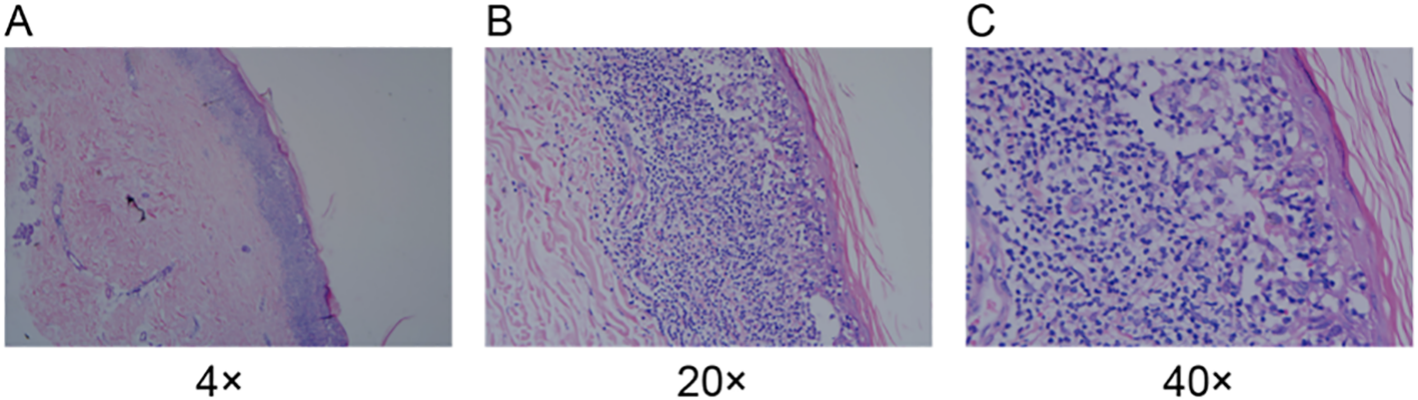
Histopathological findings of the right ankle lesion. **(A)** Low-power panoramic H&E image showing the overall architecture of the lesion, with epidermal alteration and a superficial band-like lymphocytic infiltrate beneath the epidermis, 4×. **(B)** H&E image showing prominent epidermotropism of atypical lymphocytes with a pagetoid distribution and Pautrier-like microabscesses, 20×. **(C)** High-power H&E image showing medium-sized atypical lymphocytes with pericellular halos and hyperchromatic, convoluted nuclei, 40×.

Upon physical examination, vital signs were stable. Cutaneous evaluation of the right ankle revealed a coin-sized, well-demarcated, infiltrated, scaly erythematous plaque, surrounded by scattered dusky erythematous macules with fine scale; concomitant scattered ecchymoses were observed on the left ankle (**Figure 1A**). Histopathological analysis of a punch biopsy from the margin of the right ankle lesion demonstrated mild epidermal atrophy with basal vacuolar alteration. A dense, band-like infiltrate of small-to-medium-sized lymphocytes was present in the superficial dermis, immediately beneath the epidermis, with focal epidermotropism. Within the epidermis and at the dermoepidermal junction, a population of medium-sized atypical lymphocytes displayed prominent epidermotropism with a characteristic “pagetoid” distribution and Pautrier-like microabscesses. These atypical cells exhibited pericellular halos and hyperchromatic, convoluted (cerebriform) nuclei, with occasional mitotic figures (**Figure 2**).

**Figure 3:**
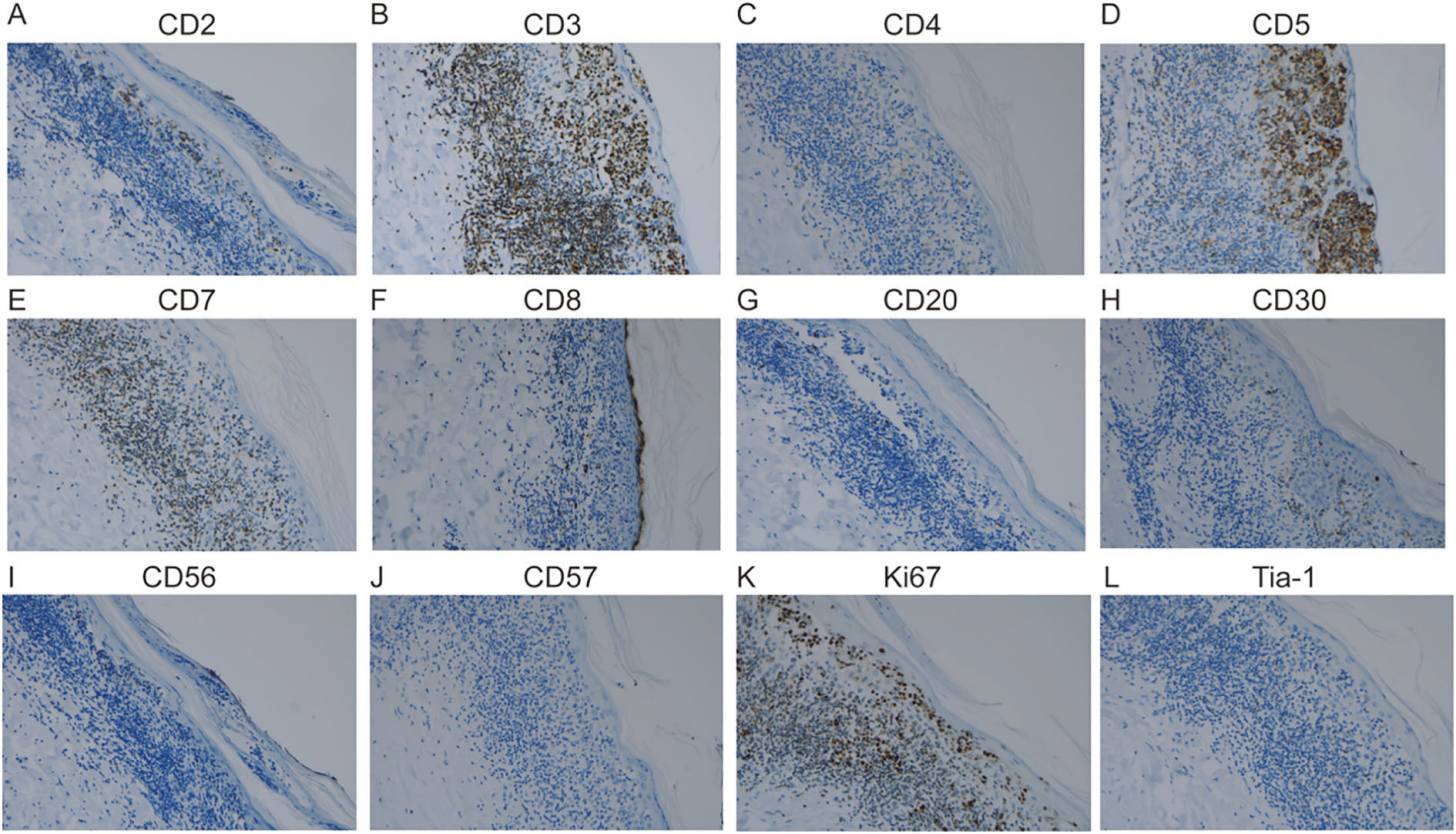
Immunohistochemical profile of the right ankle lesion. The atypical intraepidermal lymphocytes were positive for CD3 and CD5, showed loss of CD4 and CD7, and were largely negative for CD2 and CD8. Focal CD30 expression was present, and the Ki-67 proliferation index was approximately 40%. CD20, CD56, CD57, and TIA-1 were negative. **(A)** CD2; **(B)** CD3; **(C)** CD4; **(D)** CD5; **(E)** CD7; **(F)** CD8; **(G)** CD20; **(H)** CD30; **(I)** CD56; **(J)** CD57; **(K)** Ki-67; **(L)** TIA-1.

Immunohistochemical (IHC) profiling revealed that the intraepidermal atypical lymphocytes were diffusely positive for CD3 and CD5 (**Figures 3B, D**) but demonstrated a loss of CD7 and CD4 (**Figures 3C, E**). Staining for CD2 and CD8 was largely negative, with only rare positive cells identified (**Figures 3A, F**). Notably, a subset of these cells exhibited aberrant expression of CD30 (**Figure 3H**), and the Ki-67 proliferation index was calculated at approximately 40% (**Figure 3K**). In contrast, the band-like infiltrate of small lymphocytes was mainly located in the superficial dermis, immediately beneath the epidermis, and showed diffuse positivity for CD3 and CD7 (**Figures 3B, E**), with partial loss of CD8 (**Figure 3F**) and absence of CD5, CD2, and CD4 (**Figures 3A, C, D**). Both cell populations were negative for CD56, CD57, TIA-1, and the B-cell marker CD20 (**Figures 3G, I, J, L**). Additionally, EBER *in situ* hybridization was negative. This immunophenotype was compatible with a double-negative T-cell phenotype and supported the diagnosis of localized PR in the appropriate clinicopathological context.

Based on the clinical, histopathological, and immunophenotypic findings, a diagnosis of PR, a localized variant of MF, was confirmed. The patient was treated with topical 0.05% clobetasol propionate cream, applied twice daily. Because the lesion was localized and the patient preferred an initial non-invasive treatment, topical therapy was selected before radiotherapy or surgical excision. Follow-up showed substantial regression of the lesion (**Figure 1B**), and the patient remains under regular outpatient surveillance. Radiotherapy or complete excision will be considered if residual disease persists or recurrence occurs. This case highlights the need for early biopsy and immunophenotypic evaluation in patients with solitary, well-demarcated, treatment-refractory acral plaques that clinically mimic chronic eczema.

## Discussion

Under the World Health Organization–European Organization for Research and Treatment of Cancer (WHO–EORTC) classification, WKD is recognized as a rare variant of MF (2). PR is employed as a synonymous term; clinically, it is infrequent, typically manifesting as localized, slowly enlarging patches or plaques with psoriasiform or hyperkeratotic features, predominantly involving the extremities of both adults and children (9). The nomenclature is derived from the pronounced intraepidermal proliferation and epidermotropism of neoplastic T cells (10). Histopathological examination reveals psoriasiform or verruciform hyperplasia, characterized by large atypical lymphocytes scattered singly or in nests within the epidermis in a classic pagetoid pattern (7). Immunophenotypically, the Ki-67/MIB-1 proliferation index frequently exceeds 30%, and CD30 reactivity may be observed (11). While a CD8-positive phenotype predominates, CD4-positive and double-negative (CD4^-^/CD8^-^) profiles have been documented, highlighting significant immunophenotypic heterogeneity (11). Systematic reviews indicate that immunophenotypic discrepancies outweigh similarities in over half of the reported cases (12). These atypical cells originate from T-lineages: localized PR typically expresses CD4 or CD8, whereas disseminated PR frequently exhibits a double-negative phenotype and T-cell receptor (TCR) γ/δ expression (rather than α/β) with evidence of clonal rearrangement (13). Detailed clinicopathologic correlations reported since 2010 are synthesized in **Table 1**. In **Table 1**, the present case represents an additional CD4^-^/CD8^-^ localized PR case. Compared with the previously reported double-negative cases, our case showed focal CD30 positivity and a Ki-67 proliferation index of approximately 40%, without evidence of systemic involvement.

**Table 1:** Synthesis of clinicopathologic correlations reported since 2010.

No (Ref)	Age	Sex	Localization	CD4	CD8	CD30	Treatment	Year
1 (27)	63	M	Hand	–	+	ND	Topical corticosteroids +narrow-band ultraviolet B	2021
2 (16)	44	M	Leg	–	+	ND	0.05% halometasone cream + 0.02% chlormethine hydrochloride tincture	2023
3 (28)	62	F	Left wrist	–	+	+	Imiquimod	2016
4 (1)	7	M	Chest	–	+	ND	Surgery	2023
5 (7)	ND	F	Leg	–	+	+ (partial)	Surgery	2020
6 (14)	13	F	Leg	–	–	–	Clobetasol	2020
7 (29)	2	F	Hand	–	+	+ (partial)	Topical steroids/No response; Heliotherapy/partial response; Local superficial radiotherapy (20 Gy at 1.5 Gy fractions)/complete response	2018
8 (26)	10	M	Foot	ND	+	ND	PDT	2012
9 (10)	29	F	Wrists	–	–	+	Radiotherapy(3000 cGy);nitrogen mustard	2010
10 (30)	61	F	Foot	+	+ (sparse)	–	EBT	2016
11 (30)	50	M	Chest	+	+ (sparse)	ND	ND	2016
12 (3)	74	M	Foot	ND	+ (sparse)	–	ND	2019
13 (31)	52	F	Foot	+	+	ND	ND	2022
14 (25)	30	F	Hand	+	+	–	AFXL-assisted PDT	2015
15 (32)	40	M	Hand, Foot	+	–	ND	PUVA	2011
16 (33)	57	F	Forehead, ear	ND	ND	ND	Clobetasol 0.05%; nitrogen mustard; light therapy with chlormethine	2021
17 (34)	43	F	Foot	ND	+	ND	PDT	2017
18 (35)	25	F	Right upper extremity	+	+	+	Surgery	2024
19 (36)	89	M	Foot	+	ND	+ (sparse)	Ultra potent topical steroids+ psoralen+PUVA;NB-UVB	2014
20 (37)	55	F	Foot	ND	+	–	steroids	2020
21 (15)	75	M	Foot	+	+	+ (sparse)	Clobetasol proprionate ointment (0.05%)	2015
22 (18)	24	F	Foot	–	+	ND	ND	2010
23 (38)	50	F	Foot	ND	+	+	Radiotherapy	2025
24 (39)	78	M	Hand	ND	ND	ND	Alitretinoin	2013
25 (40)	53	F	Hand	+	+	–	Halobetasol cream+ bexarotene gel;bexarotene	2016
26 (41)	66	M	Arm	+	–	ND	ND	2018
27 (42)	57	F	Leg	–	+	–	Surgery	2010
28 (43)	70	M	Leg	+	–	+	PDT	2022
29 (44)	50	F	Buttock	+ (focal)	+	ND	Surgery	2014
Present case	53	F	Ankle	–	−/rare +	focal +	Topical clobetasol propionate	2026

Ref., reference; F, female; M, male; ND, not described; NB-UVB, narrow-band ultraviolet B; PDT, photodynamic therapy; RT, radiotherapy; +, positive; −, negative; partial +, partial positivity; focal +, focal positivity.

Compared with classic MF, which usually shows a CD4^+^/CD8^-^ helper T-cell phenotype, localized PR demonstrates broader immunophenotypic variability. CD4^-^ cases in PR most commonly correspond to a CD8^+^ cytotoxic/suppressor phenotype, which has been reported as the most frequent pattern. CD4^+^ cases are less common, whereas CD4^-^/CD8^-^ double-negative cases are rare. In the review by Mourtzinos et al., among 36 PR cases with available CD4 and CD8 immunophenotyping, CD8^+^ cases accounted for 53%, CD4^+^ cases for 36%, and CD4/CD8 double-negative cases for 11% (10). Although double-negative cases appeared to show a relatively higher Ki-67 proliferation index, this phenotype did not appear to confer adverse prognostic significance in localized PR.

The present case showed a CD3^+^/CD5^+^ T-cell phenotype with loss of CD4 and CD7 and largely absent CD2 and CD8 expression, corresponding to a CD4^-^/CD8^-^ double-negative phenotype. In the cases summarized in **Table 1**, this represents an additional double-negative localized PR case, increasing the number from two to three. This profile is comparable to the adult double-negative case reported by Mourtzinos et al., which showed CD7 loss, CD30 positivity, and a high Ki-67 index, although our case showed only focal CD30 expression and a lower Ki-67 index of approximately 40% (10). It also differs from the pediatric double-negative case reported by Torre-Castro et al., in which the atypical lymphocytes were mostly CD4^-^/CD8^-^ and CD30-negative, and complete remission was achieved after topical clobetasol (14).

Taken together, these findings suggest that the double-negative phenotype expands the recognized immunophenotypic spectrum of localized PR but should not by itself be interpreted as evidence of aggressive behavior. Prognosis appears to be more closely related to disease extent, localization, absence of extracutaneous involvement, and response to local therapy. Nevertheless, because CD4^-^/CD8^-^ epidermotropic T-cell infiltrates may overlap with other cutaneous T-cell lymphoproliferative disorders, careful clinicopathological correlation and close follow-up are warranted.

Compared with the more commonly reported CD8-positive phenotype in localized PR, the present case showed an unusual CD4^-^/CD8^-^ double-negative phenotype, accompanied by loss of CD2 and CD7, focal CD30 expression, and an increased Ki-67 proliferation index. This phenotype is diagnostically relevant because it broadens the recognized immunophenotypic spectrum of localized PR and may overlap with other epidermotropic variants of MF. However, CD4^-^/CD8^-^ expression alone should not be interpreted as evidence of aggressive behavior. In early MF, a double-negative phenotype has been reported as an immunophenotypic variant without clear independent prognostic significance. In localized PR, prognosis appears to be determined more by clinical extent, lesion localization, and response to local therapy than by CD4/CD8 phenotype alone. Nevertheless, this phenotype supports the need for careful clinicopathological correlation and close follow-up.

The clinical spectrum of WKD is primarily defined by indolent, asymptomatic, or mildly symptomatic isolated plaques. A systematic review comprising 84 studies and 143 patients demonstrated that the most common initial presentation was asymptomatic, slowly progressing erythema, with a mean diagnostic latency of 97.6 months (2). WKD exhibits a predilection for the limbs; these plaques may gradually expand over years, often prompting dermatological consultation only after failing conventional therapies (2, 15). The key histopathological feature is the confinement of atypical cytotoxic T-lymphocytes to the epidermis, with epidermotropism present in 97.7% of cases (2).

Differential considerations include Bowen’s disease, psoriasis, eczema, and superficial basal cell carcinoma. Owing to the lack of pathognomonic early symptoms, patients are frequently misdiagnosed with inflammatory dermatoses. Contemporary diagnostic algorithms integrate immunohistochemistry, skin biopsy, and non-invasive imaging (1–3, 14, 16). Dermoscopic findings—such as a uniform pink background with dotted or glomerular vessels and white scales—provide valuable diagnostic clues (3, 16). Furthermore, reflectance confocal microscopy (RCM) offers high-resolution, real-time visualization of lymphocytic infiltration and subtle epidermal alterations without the necessity of tissue sampling, thereby augmenting diagnostic precision (16). Although the absence of dermoscopy or RCM data in the current study constitutes a limitation, our case underscores that “well-demarcated, persistent scaly plaques on the extremities” should prompt early biopsy and integrative molecular evaluation to mitigate diagnostic delays. Diagnostic delay due to clinical resemblance to eczema or psoriasis has been widely recognized in early MF. Similarly, localized WKD may be overlooked because of its indolent course and eczematous or psoriasiform appearance. In patients with persistent, well-demarcated, treatment-refractory acral plaques, skin biopsy with immunophenotypic evaluation should be considered to support timely diagnosis.

Given the rarity of the condition, standardized treatment guidelines remain elusive, with current evidence relying largely on case series (17). Generally, localized WKD confers a superior prognosis compared to the disseminated variant. Available therapeutic modalities include surgical excision, radiotherapy (RT), photodynamic therapy (PDT), local chemotherapy, electron beam therapy (EBT), psoralen and ultraviolet A (PUVA), and topical agents (corticosteroids/imiquimod) (18–22).

RT is widely regarded as a frontline intervention due to its high efficacy, with documented doses ranging from 270 to 4300 rads (23, 24). PDT represents a safe, minimally invasive alternative that provides excellent local control while circumventing long-term radiation-induced sequelae, making it advantageous for pediatric or younger populations (25, 26). Surgical excision yields a cure rate of 86.7% in anatomically amenable areas, though challenges regarding scarring and site-specific feasibility persist (2). Notably, while high-tech interventions are effective, their cost and limited accessibility may hinder compliance, rendering topical treatments a pragmatic option for select patients.

Evidence-based comparisons of 115 patients identify RT (28.7%) and wide excision (26.1%) as the most frequent monotherapies. Data from 117 cases with follow-up indicate that RT alone achieved the most robust outcomes, with 100% complete remission and zero recurrence. In contrast, corticosteroid monotherapy demonstrated a limited cure rate of 23.1% (2). In summary, monotherapy, specifically radiotherapy, appears more effective than combination regimens for WKD (2). Nevertheless, treatment selection should also consider lesion size, anatomical site, patient preference, accessibility, cost, and tolerance of invasive procedures. In the present case, topical clobetasol was chosen as an initial non-invasive option because the lesion was small and localized, and the patient preferred conservative treatment.

Consequently, for localized, solitary lesions, RT or complete surgical excision should be prioritized. In cosmetically or functionally sensitive regions, PDT or topical immunotherapy should be considered. Continuous longitudinal surveillance is mandatory to monitor for recurrence or systemic progression.

## Data Availability

The original contributions presented in the study are included in the article/supplementary material. Further inquiries can be directed to the corresponding author.
